# Teaching implementation science in a new Master of Science Program in Germany: a survey of stakeholder expectations

**DOI:** 10.1186/s13012-017-0583-y

**Published:** 2017-04-27

**Authors:** Charlotte Ullrich, Cornelia Mahler, Johanna Forstner, Joachim Szecsenyi, Michel Wensing

**Affiliations:** Department of General Practice and Health Services Research, University Hospital Heidelberg, Im Neuenheimer Feld 130.3, 69120 Heidelberg, Germany

**Keywords:** Implementation Science, Post-graduate education, Educational research, Germany

## Abstract

**Background:**

Implementation science in healthcare is an evolving discipline in German-speaking countries. In 2015, the Medical Faculty of the University of Heidelberg, Germany, implemented a two-year full-time Master of Science program *Health Services Research and Implementation Science*. The curriculum introduces implementation science in the context of a broader program that also covers health services research, healthcare systems, research methods, and generic academic skills. Our aim was to assess the expectations of different stakeholder groups regarding the master’s program.

**Methods:**

An online survey listing desired competencies of prospective graduates was developed and administered to four groups: national experts in the field (including potential employers of graduates), teaching staff, enrolled students, and prospective students (*N* = 169). Competencies were extracted from the curriculum’s module handbook. A five-point Likert scale was used for the assessment of 42 specific items. Data were analyzed descriptively.

**Results:**

A total of 83 people participated in the survey (response rate 49%). The online survey showed a strong agreement across the groups concerning the desired competencies of graduates. About two-thirds of the listed competencies (27 items) were felt to be crucial or very important by 80% or more of participants, with little difference between stakeholder groups. Of the eight items specifically related to implementation in practice, six were in this category. Knowledge of implementation strategies (90% very important), knowledge of barriers and enablers of implementation (89%), and knowledge of evidence-based practice (89%) were the top priorities.

**Conclusions:**

The master’s program is largely orientated towards the desired competencies of graduates according to students, teaching staff, and national experts.

## Background

There is a range of educational programs on implementation science in healthcare, most of which have the format of short courses that are either stand-alone courses or are integrated into larger educational programs with a different focus (e.g., public health) [1, 2]. Implementation science remains to be a relatively new discipline and is taught at present at only a handful of universities and countries globally, most being post-graduate programs [3]. In Germany, it has only been in the past two decades that health sciences, a field covering research methodology and healthcare systems, have increasingly been integrated into undergraduate programs. In 2015, there were 43 (under-) graduate programs with a focus on health science, the majority concentrating on public health, health promotion, or epidemiology [4]. The University of Heidelberg, Medical Faculty, implemented a new two-year full-time Master of Science program “*Health Services Research and Implementation Science*” in 2015. This program is unique as it focuses specifically on healthcare delivery in all settings—hospitals, primary care, ambulatory care practices, and public health organizations. It introduces concepts and methods of implementation science, embedded in a broader curriculum that also covers health services research, healthcare systems, research methods, and generic academic skills. The background and content of the master’s program will be described below, focusing on the implementation science component of the course. The aim of the study presented in this paper was to assess the expectations of various stakeholder groups (national experts in the field, teaching staff, enrolled students, and prospective students) regarding needed competencies of prospective graduates.

### Program development

In 2011, the University of Heidelberg, Medical Faculty, introduced a bachelor program, *Interprofessional Health Care*, an innovative program enabling students to complete a bachelor degree in parallel to their vocational training in a health profession (e.g., nursing, physiotherapy). The university training of this four-year bachelor program focuses on interprofessional patient care and covers the basics of health science [5]. The success of this program as well as the shortage of graduate training in health science in Germany led to the initiative to develop a consecutive master’s program. At the same time, the Ministry of Science, Research and Arts of the state of Baden-Wuerttemberg planned to strengthen health services research. Stimulated by a federal program of this ministry to extend master’s programs in 2013, faculty members at the Department of General Practice and Health Services Research in Heidelberg developed a novel curriculum for a *Master of Science in Health Services Research and Implementation Science*. The European Framework for Higher Education—developed to ensure comparability in the standards and quality of higher education qualification within Europe—was used for guidance in the development process. Within this framework, one full-time academic year corresponds to 60 credit points (CP) equivalent to 1800 h of workload (1 CP = 30 h) [6, 7]. After formal approvals, the program started with a first cohort of 13 students in October 2015, who, by the time this article was written, are in the second year of the program. The second cohort started in October 2016 and comprises 23 students.

The master course is admission-restricted. Applicants must hold a bachelor degree related to health sciences (e.g., health care management, health economics, social sciences applied to health) or health professions (e.g., medicine, nursing, midwifery) and proof of at least basic knowledge in empirical research methods (6 CP/180 h minimum). About 20 students can be accepted each year, selected in a two-step process based on grades, practical experience, a motivation letter, and personal interviews. Languages of teaching are German and English. As a regular post-graduate and consecutive master’s program at a public university in Germany, there are no tuition fees besides the regular administration fees of approximately 150 Euro per half year. Part-time enrollment is possible.

### Program content

The master’s program in Heidelberg is designed as a stand-alone two-year full-time program with 120 ECTS in total. The curriculum focuses on five streams (see Table 1): The first stream is dedicated to *Generic Academic Skills and Research Methods* (20 CP) refreshing and enhancing the pre-existing knowledge and skills of the students with respect to the rationale, challenges, and standards of conducting research within the health care system. The second stream *Health Services Research and Health Care Systems* (22 CP) presents principles of health services research and introductions to structural and economic aspects of the health care system and health care organizations to enhance an understanding of health care. The third stream concerns *Implementation Science* (24 CP) and has a dedicated focus on the theory and practice of implementation science. A fourth stream *Fields of Application* (14 CP) supports all streams above: a yearly lecture series addresses current challenges in health care and a compulsory internship of 7 weeks offers the students work-based experiences in either providing, managing, or performing research in health care services. A complementary fifth stream concentrates on *Key Competences* (*10 CP*) such as communication or language skills, enabling students to tailor these to their individual needs and aims.

**Table 1:**
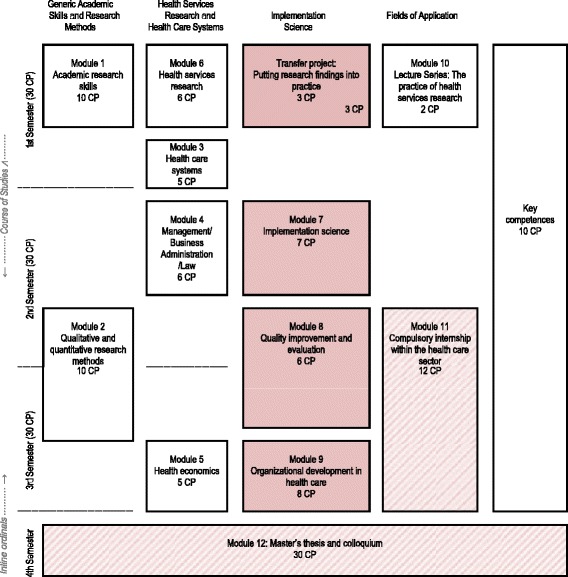
MSc Health Services Research and Implementation Science: Curriculum content in chronological order (revised version 01/2017)

1 Credit Point (CP) equals 30 h of total workload

Overall, the curriculum aims at linking four perspectives: (a) It draws from an *interdisciplinary perspective* integrating content and methods from different disciplines, such as medicine, health research, and social sciences. (b) The curriculum stresses an *international perspective* as content corresponds to both the characteristics of the German health care system and the current developments in international health services research and implementation science. (c) It identifies problems in health care and provides a better understanding of how and why implementation succeeds or fails. The perspectives of different actors—providers and users—are taken into account from an *interprofessional and patient-centred angle*. (d) The whole program is strongly research oriented, with a clear emphasis on *applied research*.

### Elaboration of the implementation science component

Overall, the master’s program has a strong focus on the implementation of evidence-based practice and improvement of healthcare delivery. Within the curriculum, four modules (24 ECTS in total) specifically focus on implementation science (as highlighted in Table 1): *Module 7 Implementation science* (*7 CP*) offers are broad conceptual foundation and orientation within the field. It introduces principles and concepts of evidence-based healthcare, provides an overview of implementation sciences as a field of research, gives insights into research studies, presents methods for systematic development and evaluation of implementation strategies, and trains skills to plan and evaluate implementation programs. *Module 8 Quality improvement and evaluation* (*6 CP*) deals with quality management in healthcare, covering quality indicators, external inpatient quality assurance, panel methods, and patient safety. Additionally, students gain knowledge on key institutions responsible for quality assurance within the German healthcare system, such as institutions of health care providers and health care politics. *Module 9 Organizational development in health care* (*8 CP*) offers insights in principles, structures, and processes of organizational development in health care institutions. It elaborates on the possibilities and constraints of change to improve health care in health care organizations. Within the *Module Transfer project* (*3 CP*) students write an outline of an exemplary healthcare intervention project discussing evidence-based health care and implementation goals and processes.

## Methods

A cross-sectional online survey was conducted to assess the views of four different stakeholder and interest groups on the desired competences of prospective graduates. The study is part of a larger study, for which approval was achieved from Heidelberg University Ethics Board (S012/2016).

### Participants/sample

A purposeful sample of four groups was planned: national experts in the field, teaching staff, enrolled students, and potential prospective students.

- The group of *national experts* in the field consists of potential employers for future graduates of the master’s program. In order to cover a broad range of relevant experts, the sample was generated by an internet-search of relevant institutions in health care delivery, health care evaluation, health policy, research and training in health systems, research and implementation science, and professional associations.
- As *teaching staff*, all core faculty that teach regularly in the master’s program were included.
- *Enrolled students* include all students enrolled in the summer term 2016, that is the first cohort of students in the master’s program.
- The group of (potential) *prospective students* represents students with a voiced interest in the program and contains three subgroups: applicants for the program starting in 2016/17, students present at an open day of the program and students of the preceding bachelor program *Interprofessional Health Care* at Heidelberg University.

### Questionnaire

A structured list of potential competences to be achieved in the program was extracted from the module handbook, which had been developed at the start of the program. The list was validated by two authors of the original module handbook (JS, CM). A draft-questionnaire was tested for clarity in face-to-face interviews. The final questionnaire covered the domains (a) health services research and health care systems, (b) implementation in practice, (c) research methods, and (d) generic academic skills. In addition, a few socio-demographic questions were added. Participants in the survey could rate the items on a five-point Likert scale ranging from: crucial, very important, somewhat important, slightly important, not important, and cannot assess.

### Procedures and data analysis

EvaSys V7.0 (2101), a software tool for surveys and training evaluation commonly used for teaching evaluations at Heidelberg University, was used to create and administer the online survey. All participants were contacted via email and completed the online questionnaire, except for the group of students attending the open day who filled in a paper-pencil version of the questionnaire. Non-responders were sent up to three reminders after 1 to 8 weeks.

Data were analyzed descriptively using SPSS Statistics 24. Frequencies of response options and percentages within each of the four groups were calculated. In a next step, percentages of the first two response options (*crucial*, *very important*) were cumulated for each group and for all groups in total. Post-hoc, we identified items with aggregated scores of 80% or higher indicating high or crucial importance as top priorities.

## Results

A total of 83 individuals provided completed questionnaires (response rate: 49%), with response rates above 40% in all groups. Prospective students were mostly female (90.2%) and mostly under 30 years of age (78%). Enrolled students were also mostly female (92%), with an average age of 32 (22–56) years. Due to data privacy, no data on age and gender of teachers and national experts in the field was collected. Most teachers (87.5%) and national experts in the field (50%) worked at universities and research institutes (Table 2).

**Table 2:** Response rates in groups and in total

	Approached (*n*)	Responders (*n*)	Response rate in %
National experts	41	18	43.9
Teaching staff	25	12	48.0
Enrolled students	12	12	100.0
Prospective students	91	41	45.1
∑	169	83	49.1

About two-thirds of the listed competencies (27 out of 42 items) were rated as crucial or very important by 80% or more of the participants. Overall, expectations of the different groups were quite similar. Some items show variations up to 35%, but the small sample size should be kept in mind (Table 3).

**Table 3:** Desired skills of graduates of the master’s program, scores 1 (crucial) and 2 (very important) aggregated

Group	National experts (*n* = 18)	Teaching staff (*n* = 12)	Enrolled students (*n* = 12)	Prospective students (*n* = 41)	∑/4	Differences between the lowest and the highest in percentage points
Expected skill						
A. Health services research and health care systems						
A1 Knowledge of key concepts	100	100	91.7	97.6	97.3	8.3
A2 Identification of relevant themes	94.4	100	91.7	97.4^b^	95.8	8.3
A3 Knowledge of structures of German health care system	94.4	100	91.7	92.7	94.7	8.3
A4 Knowledge of outcome parameters	100	91.7	91.7	95^a^	94.6	8.3
A5 Knowledge of central players of the German health care system	88.9	100	83.3	90.2	90.6	16.7
A6 Knowledge of in- and out-patient care structures	88.9	91.7	91.7	90.2	90.6	2.8
A7 Knowledge of perspectives of different actors in health care	82.4^a^	100	91.7	87.5^a^	90.4	17.6
A8 Knowledge of challenges in the German health care systems	88.9	91.7	83.3	87.8	87.9	8.4
A9 Ideas for the future developments in German health care	83.3	75	91.7	90^a^	85.0	16.7
A10 Challenges of interprofessional collaboration	72.2	91.7	75	90^a^	82.2	19.5
A11 Basic knowledge in epidemiology	77.8	75	66.7	76.9^b^	74.1	11.1
A12 Knowledge in health economics	77.8	75	66.7	75^a^	73.6	11.1
A13 Knowledge in organizational development	61.1	66.7	66.7	64.1^b^	64.6	5.6
A14 Knowledge in business administration	66.7	58.3	66.7	61	63.1	8.4
Average of all items above					84.6	10.7
B. Implementation Science						
B1 Knowledge of implementation strategies	100	91.7	75	95.1	90.4	25
B2 Knowledge of enablers and barriers in implementation	94.1^a^	91.7	83.3	87.5^a^	89.1	10.8
B3 Knowledge of evidence-based practice	88.9	100^b^	83.3	82.1^b^	88.5	17.9
B4 Knowledge of quality management in health care	83.3	90.9^a^	72.7^a^	82.9	82.4	18.2
B5 Ability to develop an evaluation plan	83.3	83.3	90.9^a^	70^a^	81.8	20.9
B6 Oral presentation skills for a public audience	83.3	83.3	83.3	73.2	80.7	10.1
B7 Expertise in evaluation of implementation plans	83.3	83.3	75	74.4^b^	79.0	8.9
B8 Work experience at health care providers	77.8	75	83.3	67.5^a^	75.9	15.8
Average of all items above					83.4	15.9
C. Research methods						
C1 Selection of appropriate research designs	88.9	100^b^	83.3	90^a^	90.5	16.7
C2 Evaluation of applied research methods	88.9	100	63.6^a^	90^a^	85.6	36.4
C3 Experience in handling different data source and routine data	88.9	81.8^a^	91.7	77.5^a^	84.9	14.2
C4 Experience in planning a research study	83.3	81.8^a^	83.3	85.4	83.4	3.6
C5 Knowledge of data sources in health reporting	77.8	100	83.3	64.1^b^	81.3	35.9
C6 Practical experience in quantitative surveys and descriptive analysis	66.7	83.3	81.8^a^	67.5^a^	74.8	16.6
C7 Knowledge of data sources for quality assessment	83.3	75	75	58.5	72.9	24.8
C8 Evaluation of study protocols	77.8	50	58.3	82.5^a^	67.1	32.5
C9 Experience in conducting qualitative interviews	66.7	58.3	83.3	60^a^	67.1	25.0
Average of all items above					78.6	22.8
D. Generic academic skills						
D1 Literature search	94.4	100^a^	75	85.4	88.7	25.0
D2 Knowledge of criteria for scientific integrity and fidelity	83.3	90.9^a^	83.3	87.8	86.3	7.6
D3 Project organization	94.4	66.7	100	82.9	86.0	33.3
D4 Oral presentations for an academic audience	100	83.3	66.7	82.5^a^	83.1	33.3
D5 Summary/review of English research literature	83.3	90.9^a^	66.7	90^a^	82.7	24.2
D6 Summary/Review of German research literature	88.9	90^b^	54.5^a^	90.2	80.9	35.7
D7 Ability to identify conflicts of interest	83.3	90.9^a^	66.7	77.5^a^	79.6	24.2
D8 Ability to write an academic report	83.3	90.9^a^	63.6^a^	73.2	77.7	27.3
D9 Knowledge of ethical and legal research guidelines	88.9	63.6^a^	75	82.9	77.6	25.3
D10 Proficiency in English	88.9	75	50	73.2	71.7	38.9
D11 Moderation of group discussions	61.1	33.3	45.5^a^	41.5	45.3	27.8
Average of all items above					78.1	27.5

^a^One missing value

^b^Two missing values

### Health services research and health care systems

All four groups largely agreed on the key topics in where they expect knowledge and skills of prospective graduates. Ten of 14 items in this domain had aggregated scores of 80% or higher, seven items reaching a score of 90% or more: “knowing key concepts,” “identification of relevant themes,” “knowing the structures of the German healthcare system,” “central players,” “structures of healthcare delivery,” and the “perspectives of various stakeholders in healthcare” (items A1–A7). The average among all groups for all items was 84.6%. Variation between the groups is less than 10% in eight of the items.

### Implementation in practice

“Knowledge of implementation strategies,” “enablers and barriers in implementation,” and “evidence-based practice” were the top priorities (B1–B3). The average among all groups for all items was 83.4%. Within the eight items in this domain, differences vary from 8.9 to 25% between groups (average, 15.9%).

### Research methods

The top priorities in this domain were “knowledge of study designs,” “applied research methods,” “data sources,” “routine data,” and “planning of a study” (C1–C5). The average among all groups was 78.6% for aggregated rating. Within the nine items, differences vary from 3.6 to 36.4% (average, 22.8).

### Generic academic skills

Six of the 11 items had a rating of 80% or more: “literature search,” “scientific integrity,” “project organisation,” “oral presentation skills,” and “making summaries and reviews of the English and German literature” (D1–D6). The average among all groups was 78.1% for aggregated rating. Within all items, differences vary from 7.6 to 38.9% (average, 27.5).

## Discussion

The newly established Master of Science program *Health Services Research and Implementation Science in Healthcare* addresses the needs of students, teaching staff, and national experts in the field (including potential employers of graduates). While some differences between groups were found, we are careful with interpretations given the relatively small sample size. Though not statistically significant, observable diverging expectations on research skills and academic ethos on the one hand and applied orientations on the other hand reflect the experiences of the teachers of the master’s program: Most students are more interested in topics that are directly linked to practical problems of the health system than more general aspects of academic training. Teachers, in contrast, set high priority on ability to critically assess different data resources and research methods as well as general academic aspects such as scientific integrity and fidelity and competences in literature reviews and academic writing. This difference might be attributed to some extent to the setup as an academic program of a two-year master’s. In a few respects, the expectations of enrolled students match those of the teaching staff more than those of the prospective students. This might hint to an assimilation and adaption process during the first year of study.

For graduates and advanced post-doctoral training in the science and practice of knowledge transfer (KT) research—roughly equivalent to implementation science—by Straus et al. [8] identified the following core competencies: (a) knowledge and understanding of models and theories of KT and KT research, (b) capacity to conduct syntheses or address KT questions, (c) capacity of multiple research methods to examine the determinants of knowledge use across settings and stakeholder groups, and (d) capacity to design and evaluate impact, effectiveness, and sustainability of KT strategies in different settings. A study using input of dissemination and implementation research experts to develop a list of competencies in the field by Padek et al. [9] identified the four domains of (a) definition, background, and rational; (b) theory and approaches; (c) design and analysis; and (d) practice-based considerations. The competencies in both studies correspond closely with content in the *domain A* “key content and research perspective” and domain C “research methods” of the master’s program in Heidelberg. In addition, the domains B “implementation in practice” and D “generic academic skills” relate to the first domains of Padek et al.

Surveys on (potential) trainees mirrored these results to a certain degree [1, 10, 11]. In an online survey on knowledge transfer trainees’ priorities in Canada, Newman et al. [10] identify seven main themes, among them importance of competencies in context, theory and methods. Stamatakis et al. [1] highlight training and background in a variety of methods as crucial for a group of initial implementation science trainees in the USA that are involved in an implementation research training program. However, both studies also underline different priorities such as the use of technology like the web and health informatics [10] and developing practice linkages [1].

## Conclusions

The presented online survey provides a snapshot of the views of various stakeholders in Germany on the competencies needed in the field. It shows that the discussed master’s program is largely orientated towards the desired competencies of graduates according to students, teaching staff, and national experts. However, the response rate and focus on Baden-Wuerttemberg (a region in Germany with 11 million inhabitants) should be kept in mind. Future research is planned to comprise qualitative interviews, distinguish different groups of experts (such as health care providers, political actors, and researchers), and include the alumnae of the program. This research will focus on the outcomes of the master’s program and discuss recommendations for the field, an area which cannot be addressed at this stage of this innovative program. This master’s program will generate a substantial number of individuals with post-graduate training in health services research and implementation science. In contrast to supplementary post-graduate short training programs that build on actual experience in implementation, the master’s program aims at a broad academic training for students with a bachelor’s degree related to health sciences that might include practical training in a health care profession.

Different expectations of academic teaching staff on the one hand and stakeholders outside academia on the other hand lay in the nature of their perspectives; these differences are neither unpreventable nor necessarily obstructive to improve care. However, it is the distinct object of the program to generate graduates who have the knowledge and skills to contribute to narrowing the gap between research and practice in health care. Most students have—at least in the beginning—a stronger interest in practical tasks rather than theoretical underpinnings, research methods, and fundamental academic ethos. However, in our opinion, the last three are required to understand and explain how and why implementations succeed or fail. One of the major challenges for the further development of curriculums will be to put an even stronger focus on expatiating on these connections of research and practice.

It is difficult to assess the need for health researchers in a country, but it seems fair to say that the numbers are small in Germany compared to some other countries. We regard the implementation of the master’s program as a wise investment for the Federal State of Baden-Wuerttemberg regarding health-related education and research. This will pay off in the coming decade when its graduates have entered the healthcare system and successfully tackle the challenges of the future.
